# Distinct Distal Gut Microbiome Diversity and Composition in Healthy Children from Bangladesh and the United States

**DOI:** 10.1371/journal.pone.0053838

**Published:** 2013-01-22

**Authors:** Audrie Lin, Elisabeth M. Bik, Elizabeth K. Costello, Les Dethlefsen, Rashidul Haque, David A. Relman, Upinder Singh

**Affiliations:** 1 Department of Microbiology and Immunology, Stanford University School of Medicine, Stanford, California, United States of America; 2 International Centre for Diarrhoeal Disease Research, Bangladesh, Dhaka, Bangladesh; 3 Department of Medicine, Stanford University School of Medicine, Stanford, California, United States of America; 4 Veterans Affairs Palo Alto Health Care System, Palo Alto, California, United States of America; Cairo University, Egypt

## Abstract

**Background:**

Our current understanding of the composition and stability of the human distal gut microbiota is based largely on studies of infants and adults living in developed countries. In contrast, little is known about the gut microbiota and its variation over time in older children and adolescents, especially in developing countries.

**Methodology/Principal Findings:**

We compared the diversity, composition, and temporal stability of the fecal microbiota of healthy children, ages 9 to 14 years, living in an urban slum in Bangladesh with that of children of the same age range in an upper-middle class suburban community in the United States. We analyzed >8,000 near full-length 16S rRNA gene sequences and over 845,000 pyrosequencing reads of the 16S rRNA V1–V3 region. The distal gut of Bangladeshi children harbored significantly greater bacterial diversity than that of U.S. children, including novel lineages from several bacterial phyla. Bangladeshi and U.S. children had distinct fecal bacterial community membership and structure; the microbiota of Bangladeshi children was enriched in *Prevotella*, *Butyrivibrio*, and *Oscillospira* and depleted in *Bacteroides* relative to U.S. children (although similar to Bangladeshi adults). Furthermore, community membership and structure in Bangladeshi children was significantly less stable month-to-month than U.S. children.

**Conclusions/Significance:**

Together, these results suggest that differing environmental or genetic factors may shape the microbiota of healthy children in the two countries. Further investigation is necessary to understand the mechanisms and factors that underlie these differences, and to incorporate these findings into new strategies for the prevention and treatment of childhood and adolescent diseases.

## Introduction

Of the 1.2 billion global adolescent population, 88% live in developing nations where the incidence of environmental enteropathy – a multifaceted, subclinical intestinal disorder encompassing repeated episodes of infectious gastroenteritis, chronic inflammation, and malnutrition – ranges from 50–95% [1], [2], [3]. It is thought that the human indigenous gut microbiota may potentially serve a critical role in this disorder [4]. To date, the human gut microbiota has been characterized in depth using molecular approaches from individuals in only a few low-income areas of the developing world [5]. Recent work highlights the importance of geography in explaining the gut microbiota composition of adults and children [6], [7] and underscores the need to select additional geographic settings in an effort to characterize the global extent of human-associated microbial diversity [8]. Furthermore, the influence of geography on the temporal stability of the composition of the gut microbiota within healthy individuals has not previously been examined, in part because of the difficulty in obtaining serial samples from a consistently healthy reference population in areas with high rates of environmental enteropathy or other gastrointestinal conditions.

Long-term monitoring programs such as those at the International Centre for Diarrhoeal Disease Research, Bangladesh (ICDDR,B) present a valuable opportunity to select samples in a retrospective manner from healthy at-risk children without recent disease (http://report.nih.gov/; NIH grant 5R01AI043596). The present study compares the taxonomic composition of the distal gut microbiota of relatively healthy children, ages eight to fourteen, living in an urban slum in Bangladesh with that of children of the same age range living in an upper-middle class suburban community in the United States. We used a cultivation-independent, molecular phylogenetic approach, relying on both near full-length and shorter hypervariable region 16S rRNA sequences, to compare the monthly intra-individual dynamics and interpersonal variation of fecal microbial communities in Bangladeshi and U.S. children.

## Materials and Methods

### Sample collection

Fecal specimens from 6 Bangladeshi children (ages 8–13), 4 Bangladeshi adults (ages 18–41), and 4 U.S. children (ages 10–14 years) were selected for study from a much larger population of subjects and associated specimens. The fecal sampling schedule consisted of 5 consecutive monthly samples from 4 of the Bangladeshi children, 6 consecutive monthly samples from the U.S. children, and 1 sample each from the Bangladeshi adults and the other 2 Bangladeshi children (Table 1).

**Table 1:** Characteristics of the participants in this study.

Subject ID	Sex	Age	Country	Ethnicity	Samples Analyzed (Collection Month)					
					1	2	3	4	5	6
BC9F	F	9	Bangladesh	Bihari	Nov	Dec	Jan	Feb	Mar	
BC13F	F	13	Bangladesh	Bihari	Jan*	Feb*	Mar*	Apr*	**May** *	
BC12M	M	12	Bangladesh	Bihari	Dec*	Jan*	Feb*	Mar*	Apr*	
BC13M	M	13	Bangladesh	Bihari	Apr*	**May** *	**Jun** *	**Jul** *	**Aug** *	
UC12F	F	12	U.S.A.	Chinese	Feb	Mar	Apr	May	Jun	Jul
UC14F	F	14	U.S.A.	Chinese	Feb	Mar	Apr	May	Jun	Jul
UC10M	M	10	U.S.A.	European	Feb	Mar	Apr	May‡	Jun	Jul
UC13M	M	13	U.S.A.	Chinese	Feb	Mar	Apr	May	Jun	Jul
BK8F	F	8	Bangladesh	No Data	**May** †					
BK9F	F	9	Bangladesh	No Data	**May** †					
BA35F	F	35	Bangladesh	No Data	Nov†					
BA25M	M	25	Bangladesh	No Data	Jan†					
BA33M	M	33	Bangladesh	No Data	Dec†					
BA41M	M	41	Bangladesh	No Data	**Aug** †					

* **Additional near full-length sequences were generated for these 15 samples.**

† **To control for potential barcode effects, duplicates of these DNA samples were tagged with different barcodes.**

‡ **Biological replicate: two portions derived from the same fecal sample underwent two separate extraction procedures.**

16S rDNA V1–V3 pyrosequencing reads were generated for all of the samples. Monsoon months for Bangladeshi samples are highlighted in bold. Bangladeshi child 12 M was stunted with a height-for-age Z-score (HAZ) of −2.42. Due to the constraints of previously-designed studies, the BK and BA participants met different exclusion criteria than the UC and BC participants. BK and BA participants did not have diarrheal illness for 3 months or anti-parasitic medicine for 1 month before the first fecal sample. In contrast, the UC and BC participants reported no diarrheal illness, received no antimicrobial therapy, and had no identified enteric pathogens for 3 months prior to the first fecal sample.

### Human subjects

Three groups of subjects were studied (Table 1).

#### 1) Bangladeshi children

Since 1999, Haque *et al* have conducted a longitudinal study of susceptibility to amebiasis and enteric infections and have amassed a study cohort in Section 11 of the densely populated Mirpur urban slum of Dhaka, which has ∼50,000 residents. Most of the residents are of Bihari ethnic origin and remain mired in low socioeconomic conditions with an average monthly income per family of <5,000 takas (U.S. $61). 75% of fathers and 85% of mothers have less than five years of education, and rice and bread are the main dietary staples of the daily diet. Unsafe water, inadequate sanitation, and poor hygiene are the main conduits for disease transmission in Mirpur. Only 33% of the households have direct access to water from a municipal supply with the remaining households obtaining water through plastic pipes that illegally siphon municipal water but are often cracked and contaminated and located near latrines and drains. Residents use sanitary latrines or pit latrines that are discharged into sewage flowing openly through the slum.

A retrospective analysis was conducted on fecal specimens from this existing cohort of ∼420 children ages 8–13. ICDDR,B health care workers visited and interviewed the children and their families at their homes on alternating days to monitor health and stool characteristics (frequency, presence of blood, accompaniment by fever). Patient medical history, socioeconomic characterization, house location, and nutritional status were recorded by the ICDDR,B field team. Episodes of diarrheal illnesses and associated diagnosis and treatments were also recorded for each child. A monthly surveillance non-diarrheal fecal specimen was collected from each child. Field staff instructed caregivers to ask the child to defecate on a plastic sheet and to collect material from the top of a fresh stool pile that had not contacted any other surface. All fecal specimens were stored in stool collection vials and transported on ice to ICDDR,B within 2–4 hours after collection. Fecal samples were stored at −80°C. Stools were screened for bacteria (including enterotoxigenic *Escherichia coli*, enteropathogenic *E. coli*, *Shigella flexneri*, *Campylobacter jejuni*, *Salmonella* sp., *Vibrio cholerae*, *Aeromonas hydrophila*, *Plesiomonas shigelloides*), viruses (rotavirus, astrovirus, adenovirus), and parasites (*Entamoeba histolytica*, *Cryptosporidium parvum*, *Giardia lamblia*, *Trichuris trichiura*, *Ascaris lumbricoides*, *Strongyloides stercoralis*, *Hymenolepis nana*, and hookworm) using standard culture methods, microscopy, and antigen detection tests (see [9] for the complete list of tested agents and detailed methods). Based on these data, a subset of 4 healthy children was selected who had received no antimicrobial therapy and whose stool had no identified enteric pathogens for 3 months prior to the first sample selected for analysis in this study and for the following 5 consecutive months (8 consecutive months in total).

#### 2) Adults and additional children from Bangladesh

A single fecal sample was collected from 4 adults (ages 18–41) and 2 children (ages 8 and 9) from various locations in Dhaka who were control subjects enrolled in a case-control enteric disease study through the outpatient clinics of the Bangabandhu Sheikh Mujib Medical University [10]. Standard hospital protocols for collecting fecal specimens were followed. The adults and children reported no diarrheal illness or ingestion of anti-parasitic medicine for 3 months and 1 month before sample collection respectively. For these subjects, no specific information was available on antibiotic use. All fecal samples tested negative for parasitic agents including *Entamoeba histolytica*, *Cryptosporidium parvum*, *Giardia lamblia*, *Trichuris trichiura*, *Ascaris lumbricoides*, and hookworm with microscopy and antigen detection methods. Ethnicity and socioeconomic data were not collected for these individuals.

#### 3) U.S. children

Six consecutive monthly fecal samples were collected from 4 healthy children living in northern California and Oregon. These children met the following criteria: no antibiotics, diarrheal illnesses, international travel, or known bacterial, viral, or parasitic disease for 3 months prior to the first collected sample and during the 6-month collection period. Parents were given instructions for sample collection using fecal collection containers with integrated measuring spoons on their lids (Sarstedt Inc., Newton, NC). After collection, fecal samples were immediately stored at −20°C. Within 24 hours, fecal samples were transferred on ice to the laboratory and stored at −80°C.

### Ethics Statement

Bangladeshi adult and child fecal specimens were obtained as part of a previous study that followed the human experimentation guidelines of the U.S. Department of Health and Human Services, the University of Virginia, and the Centre for Health and Population Research, ICDDR,B [9]. The parents of the U.S. children gave written informed consent in accordance with a protocol approved by the Stanford University Administrative Panel on Human Subjects in Medical Research.

### DNA extraction

The QIAamp® DNA Stool Mini Kit (Qiagen, Inc., Valencia, CA) was used to extract DNA from 200 mg of feces according to the manufacturer's instructions. DNA was eluted in a final volume of 200 µl elution buffer and stored at −80°C. Tubes without fecal material were processed in parallel to serve as negative extraction controls.

### Amplification, quantitation, and pyrosequencing of variable regions 1–3 of the 16S rRNA gene

V1–V3 rDNA pyrosequencing was performed on all samples from U.S. children and Bangladeshi children and adults (Table 1). The V1–V3 region (∼490 nt) was amplified with a primer set previously described [11]. A 5 or 50 ng aliquot (variation was negligible between 1∶10 dilution or undiluted sample replicates tested in [11]) of each extracted DNA sample served as the template for 50 µl PCRs, which contained 1X FastStart High Fidelity reaction buffer with MgCl_2_ (Roche), 200 µM deoxyribonucleoside triphosphates, 200 nM of the proximal fusion primer, 280 nM of the distal fusion primer, and FastStart High Fidelity enzyme blend (5 U/reaction; Roche). Thermal cycling consisted of 3 min at 94°C, and 25 cycles consisting of 30 s at 94°C, 45 s at 51°C, and 5 min at 72°C, followed by 10 min at 72°C. Gel electrophoresis was used to visualize the PCR products, and products from two to four replicate amplification reactions were pooled for each sample. PCR amplicons were gel purified and the DNA concentrations were quantified using the Quant-iT™ PicoGreen® dsDNA reagent and kit (Invitrogen, Carlsbad, CA) and a Typhoon scanner (GE Healthcare, Piscataway, NJ). Following quantitation, the amplicons were pooled in equimolar ratios and sequenced using the 454 Life Sciences Genome Sequencer FLX Titanium platform (Roche, Branford, CT).

### Quality filtering of pyrosequencing reads

We processed 1,140,964 raw reads using the Quantitative Insights Into Microbial Ecology (QIIME) software package [12]. Preprocessing involved removing reads lacking a barcode or primer sequence, and then removing the forward and reverse primer sequences from the reads. Sequences with a length <200 nt or >580 nt were excluded. A maximum of 1 ambiguous base per read was allowed. Sequences were discarded if the average quality score over a sliding window spanning 50 nucleotides dropped below 25. Sequences containing a homopolymer run exceeding 6 nt were rejected. To facilitate the further removal of erroneous reads, the remaining 850,782 quality-filtered sequences were denoised using QIIME Denoiser [13].

### Modification of an existing reference 16S rRNA database

Pyrosequencing reads were compared to a reference database with UCLUST software [14]. To create a high-quality reference database with broad phylogenetic coverage of and shared similarity with our query sequences, we supplemented the Greengenes near full-length reference database (gg_99_otus_4feb2011_aligned.fasta; 84,566 unique operational taxonomic units (OTUs)) with the 503 near full-length OTU representative sequences from the 3 Bangladeshi children described below (see Methods section “*Amplification, cloning, and sequencing of near full-length 16S rRNA genes*”). To avoid redundancies in the final reference database, we clustered these 503 representative sequences and the Greengenes reference set with UCLUST at a similarity threshold of 97%. The 153 sequences that failed to cluster at this threshold setting were combined with the Greengenes reference sequences to form a combined Greengenes-Bangladesh reference database. This new Greengenes-Bangladesh reference database was aligned against the Greengenes core set alignment and a modified PH Lane mask was applied to trim the sequences to the V1–V3 region and then clustered at a 99% similarity threshold. The most abundant sequence within each OTU was selected as the OTU representative sequence. The resulting final Greengenes-Bangladesh reference database contained 63,774 representative OTUs. To ensure that our modification of the Greengenes reference database did not bias the subsequent OTU clustering steps, we performed *de novo* clustering with and without the unmodified Greengenes reference database and obtained results similar to the modified reference database approach (data not shown).

### Analysis of quality-filtered V1–V3 pyrosequencing reads

For the pyrosequencing data set, an OTU was defined at 97% sequence similarity (in contrast to the 99% OTU definition for near full-length Sanger sequences) because thresholds more stringent than 97% for pyrosequencing reads tend to inflate diversity estimates [15]. Pyrosequencing reads within the 97% similarity threshold were identified with UCLUST (800,465 query reads binned into 1,831 reference OTUs). 45,205 query reads that failed to cluster with reference sequences at this similarity threshold were binned into 2,609 new OTUs. The most abundant sequence within each new or reference OTU was chosen as the OTU representative sequence. Because novel sequences were discovered in the Bangladeshi near full-length dataset (as described below), one benefit of using this hybrid reference database and *de novo* clustering approach was that OTU clusters were first seeded with high-quality reference sequences; the *de novo* clustering allowed the binning of novel reads (instead of discarding them). The PyNAST alignment algorithm [16] was used to align OTU representative sequences against the Greengenes core set alignment with a minimum alignment length of 150 and a minimum identity of 75% (35 OTU representative sequences failed to align). The OTU representative sequences were screened for chimeras with the Chimera Slayer algorithm and the BLAST fragments approach in QIIME, and the intersecting set of flagged sequences from both methods (1,848 representative sequences) were excluded from the alignment and downstream analysis. The PH Lane mask was applied to the alignment to retain the conserved regions of the 16S rRNA gene and omit the hypervariable regions for phylogenetic inference. Based on this alignment of OTU representative sequences, a phylogenetic tree was inferred using FastTree software [17]. Taxonomy was assigned to each OTU representative sequence using the Ribosomal Database Project (RDP) classifier trained on the Greengenes full-length reference database (gg_99_otus_4feb2011_aligned.fasta) [18], [19]. The taxonomic assignment of each sequence was truncated at the most specific taxonomic level with a confidence score of at least 80%.

### 16S rDNA V1–V3 sequence diversity analysis

Good's estimator of coverage, the proportion of non-singleton OTUs in the dataset as a measure of overall OTU sampling completion, was used to assess the adequacy of sampling. Microbial diversity was evaluated within samples (α diversity) or between samples (β diversity) using QIIME. Rarefaction, to a subsampling depth (determined by the minimum number of sequences in a sample from a single time point) of 6,596 reads/sample (unless otherwise noted) and 5 iterations, was performed on all samples to standardize the sequencing effort. Alpha diversity was measured with the Chao1 (richness), Phylogenetic Diversity (branch length-based diversity [20]), Shannon entropy (OTU-based diversity), and observed species metrics. Alpha diversity means and statistical significance tests were calculated in Excel and error bars represent standard error of the mean (SEM). Beta diversity was evaluated with UniFrac, a community dissimilarity metric based on the fraction of unique branch length observed in pairs of communities in a common phylogenetic tree [21], [22]. The phylogenetic distance (UniFrac distance) is calculated as the fraction of unshared branch lengths between the pair of communities. Unweighted and weighted UniFrac distance matrices served as inputs for clustering analyses and significance tests aimed at determining which variable explained the greatest amounts of variation in the taxonomic composition of the gut bacterial communities [23].

Unweighted UniFrac distances compare microbial communities within a phylogenetic context based on the presence/absence of members, while weighted UniFrac also incorporates relative abundance information [23]. UniFrac-based Principal Coordinate Analysis (PCoA) aids in the exploration of potential factors (such as country or age) that might explain the groupings of similar communities. PCoA, visualized with KiNG software (http://kinemage.biochem.duke.edu/software/king.php), was used to map the UniFrac distance matrix onto a set of orthogonal axes capturing the greatest amount of variation in all the samples tested. Distances between samples on a PCoA plot reflect the corresponding dissimilarities in their community membership (unweighted UniFrac) or community structure (weighted UniFrac). Samples were hierarchically clustered based on their inter-sample UniFrac distances using Unweighted-Pair Group Method with Arithmetic mean (UPGMA), and jackknifing support was provided at each internal node by re-sampling 100 times with replacement at a depth of 6,596 sequences per sample.

Statistical significance of factors potentially contributing to compositional differences among microbiota samples was tested with PRIMER software using the non-parametric permutation analysis of similarity (ANOSIM) function, analogous to the univariate ANOVA (analysis of variance) test [24]. The ANOSIM global test statistic, R, generally returns a value between −1 and 1. R approximates 0 if no differences are observed according to sample class (e.g., country of origin), and R = 1 if all samples within a given class are more similar to each other than samples from a different class. For comparative purposes, nine U.S. adult samples from three previous studies [11], [25], [26] were included in beta (but not alpha) diversity analyses; differences between studies in DNA extraction and sequencing methods have a large influence on measures of alpha diversity but relatively small effects on beta diversity metrics. Re-sampling-based multiple testing was performed using the ‘mt’ function in the Phyloseq package of R (which calls the ‘mt.maxT’ function in the multtest multiple testing package in R) [27]. The V1–V3 16S rDNA bacterial sequences analyzed in this paper have been deposited in the GenBank Short Read Archive (Accession number: SRA057705).

### Amplification, cloning, and sequencing of near full-length 16S rRNA genes

Near full-length 16S rRNA gene surveys were performed on the 5 consecutive monthly samples from each of the following 3 Bangladeshi children: BC12M, BC13M, and BC13F (Table 1). The 16S rRNA gene was amplified using two broad-range bacteria-specific primers: Bact-8F (5′-AGAGTTTGATCMTGGCTCAG-3′) [28], [29] and Bact-1391R (5′-GACGGGCGGTGTGTRCA-3′) [29], [30]. These primers amplify more than 90% of the 16S rRNA gene (∼1400 nt). PCR was performed as previously described [25], with the addition of 2% dimethyl sulfoxide. Thermal cycling settings were as follows: 5 min of initial denaturation at 95°C, 20 cycles consisting of 30 s of denaturation at 94°C, 30 s of annealing at 55°C, and 90 s of elongation at 72°C, followed by 8 min of final extension at 72°C. Gel electrophoresis of 5.0 µl of PCR product on 1.0% agarose gels was used to confirm the size of the products for the 15 Bangladeshi child samples. For each sample, four replicate 20-cycle PCRs were performed and the amplicons pooled to obtain adequate product yields for cloning. Gel electrophoresis did not reveal visible PCR products from any of the DNA extraction controls or the negative PCR controls. Amplicons were purified with the QIAquick® PCR Purification Kit (Qiagen), eluted in a final volume of 40 µl elution buffer, stored at −20°C, and shipped on dry ice to the J. Craig Venter Institute (Rockville, MD) for cloning and Sanger sequencing.

### Phylogenetic analysis of near full-length 16S rRNA sequences

Paired-end Sanger sequencing reads from cloned PCR products were edited and assembled into 8,158 near full-length 16S rRNA gene sequences as previously described [25]. Assembled sequences were aligned against the Greengenes core set using the NAST algorithm [31] (http://greengenes.lbl.gov) and then imported into ARB (version 08.08.27; [32]) for phylogenetic analysis. In ARB, the alignment was manually improved using secondary structure information and alignment to close relatives. Ninety-three poor quality sequences and 26 chimeras (0.3% of the sequences) were identified and removed by hand, and the complete dataset was also screened for chimeras using Mallard (version 12.2.0; [33]). Upon further manual assessment, none of the additional sequences flagged by Mallard was believed to be chimeric. The 8,039 high-quality sequences were grouped into 503 OTUs using a 99% sequence identity cut-off after applying a modified Lane mask (1256 nt) to screen out hypervariable regions, as previously described [25]. A stringent 99% similarity phylotype threshold was chosen because bacteria with masked, near full-length 16S rRNA sequences that display more than 1% dissimilarity may represent distinct species [34], [35]. A neighbor-joining tree was inferred from an Olsen-corrected distance matrix. The DOTUR and mothur packages were used to calculate Chao1 and collectors' curves [36], [37]. Taxonomy was assigned using the Greengenes database. Sequences without close Greengenes neighbors in ARB were queried against the NCBI GenBank database using the basic local alignment search tool (BLAST) algorithm. The near full-length bacterial 16S rRNA gene sequences generated in this study have been deposited in GenBank (Accession numbers: JQ183111–JQ191149).

## Results

### Characteristics of the Bangladeshi and U.S. participants

We compared the distal gut microbiota of four healthy Bangladeshi children (ages 9–13 years) living in the Mirpur urban slum of Dhaka to that of four healthy U.S. children (ages 10–14 years) from affluent regions of California and Oregon. Despite a high incidence of helminth infections and diarrheal diseases in the urban slum conditions of the existing ICDDR,B cohort of ∼420 children, six children (including the four we studied – Bangladeshi children 9F, 13F, 12M, and 13M) remained healthy and free of identified bacterial, viral, and parasitic infections for eight consecutive months. Rice, bread and lentils are the main staples of their daily diet. Five consecutive monthly samples were obtained from each of the four Bangladeshi children, and six consecutive monthly samples were obtained from each of the four U.S. children (Table 1). One sample each from four unrelated healthy Bangladeshi adults (ages 18–41 years) and two additional healthy Bangladeshi children (ages 8–9 years) from various sites within Dhaka were also included in the analysis. The age-matched U.S. children (3 Chinese-Americans, 1 European-American) lived in upper-middle class households and consumed typical Western diets. Compared to the daily diet of the Bangladeshi children, the typical diet of the U.S. children was more diverse and included carbohydrates, vegetables, and various sources of animal protein.

### Community richness and biodiversity of the healthy distal gut

The V1–V3 hypervariable regions of the 16S rRNA gene were PCR-amplified from each of the 54 fecal samples collected from the 14 subjects, and 845,670 high-quality pyrosequencing reads were generated in total (6,596–28,404 per sample, Table 2) with an average read length of 485 nt. These reads were clustered at a 97% similarity threshold into 4,440 unique OTUs (156–1,126 per subject). The Chao 1 estimated richness of the entire dataset was 8,575 OTUs (228–2052 OTUs per participant, Figure S1A). Good's coverage averaged 99.61% in the Bangladeshi children (BC) and 99.82% in the U.S. children (UC). These V1–V3 reads were assigned to 348 genera (52–227 genera per participant) and 19 phyla (7–17 phyla per participant). Our data also included 8,039 near full-length, non-chimeric sequences from 15 fecal samples of 3 Bangladeshi children (5 consecutive monthly fecal samples per child, Table 1) (average of 536±104 (SD) sequences per sample) representing 503 unique OTUs (clustered at a 99% similarity threshold).

**Table 2:** Summary of sequencing, OTUs, coverage, and taxa.

Subject	Full-Length Sequences	V1–V3 Reads	Observed OTUs_0.97_	Estimated OTUs_0.97_ *	Good's Coverage	Phyla	Genera
BC9F		62813	870	1330	99.63	10	172
BC13F	2878	87905	1126	2052	99.56	13	170
BC12M	2512	81554	957	1488	99.70	17	227
BC13M	2649	57035	906	1376	99.54	11	175
UC12F		93208	540	865	99.86	9	90
UC14F		83483	572	994	99.82	8	106
UC10M		110098	909	1827	99.72	9	132
UC13M		120649	587	1039	99.87	8	101
BK8F		27793	414	575	99.57	9	106
BK9F		26761	156	228	99.82	6	52
BA35F		19241	392	484	99.52	8	104
BA25M		23707	305	436	99.61	11	105
BA33M		28311	655	917	99.30	8	111
BA41M		23112	361	469	99.60	7	96
**Total**	**8039**	**845670**	**4440**	**8575**	**99.82**	**19**	**348**

* Estimated OTU richness calculated using the Chao1 metric.

Abbreviations: OTUs_0.97_, Operational Taxonomic Units defined at a<97% similarity threshold. Only the V1–V3 16S rDNA pyrosequencing data (pooled time points for each BC or UC participant) were used to calculate Observed OTUs_0.97_, Estimated OTUs_0.97_, Good's coverage, Phyla, and Genera. Values in the table cannot be compared across individuals because the counts shown here are not rarified to reflect the extent to which the gut microbiota has been sampled by V1–V3 16S rDNA pyrosequencing. Good's estimator of coverage was calculated per individual using the formula: [1 - (n/N)] * 100% where n is equal to the number of singleton reads and N is the total number of reads.

### Microbiota from Bangladeshi children is more diverse than microbiota from U.S. children

**Figure 1:**
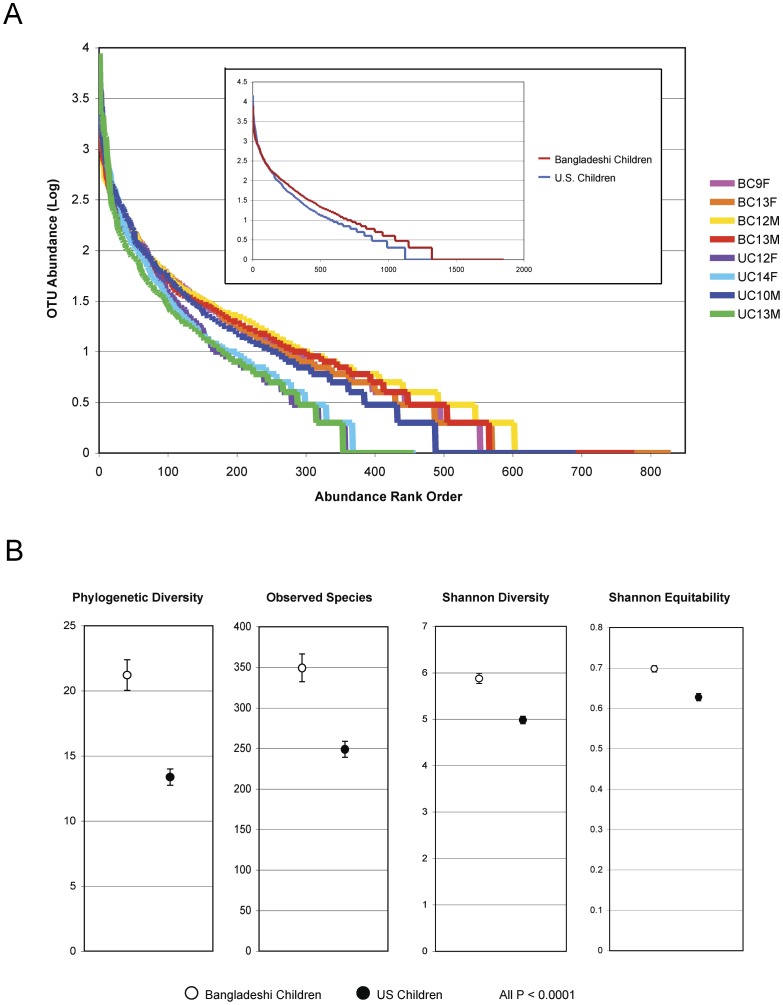
Alpha diversity based on 16S rDNA V1–V3 sequence data. (A) Rank abundance curves. The vertical axis displays OTU relative abundance on a logarithmic scale. The horizontal axis lists the OTUs in rank order of descending abundance. (B) Alpha diversity statistics. Phylogenetic Diversity (PD) is the minimum total branch length of the phylogenetic tree that incorporates all OTUs in a sample. The number of observed species was calculated at a similarity threshold of 97%. Shannon Diversity index (H) characterizes species diversity and accounts for abundance and evenness of the species. Shannon equitability index (E_H_) is a measure of evenness. If S is the number of observed species, then E_H_ = H/ln (S). Due to the unequal number of measurements in the time series for each child (5 for Bangladeshi children and 6 for U.S. children), all 5 monthly measurements from each of 4 Bangladeshi children and the first 5 monthly measurements from each of 4 U.S. children were averaged. Only samples from the children (BC and UC participants) were included in this alpha diversity analysis (Table 1). Error bars represent the standard error of the mean (SEM).

After rarefaction to 6,596 reads per sample, comparisons of the V1–V3 sequences using several alpha diversity metrics revealed significant differences between the two groups of subjects. Overall, the Bangladeshi children exhibited greater diversity, including greater evenness, than the U.S. children, as revealed by the individual rarefaction and rank abundance curves (Figure 1A and Figure S1B) and the Shannon Equitability Index (0.70 ± 0.01 for Bangladeshi children and 0.63 ± 0.01 for U.S. children; P<0.0001, two-tailed t-test with unequal variance; Figure 1B). Bangladeshi children were characterized by significantly higher levels of phylogenetic diversity (21.2 ± 1.2 in Bangladeshi children versus 13.4 ± 0.6 in U.S. children; P<0.0001; Figure S2), higher Shannon entropy (5.9 ± 0.1 in Bangladeshi children versus 5.0 ± 0.1 in U.S. children; P<0.0001), and a larger number of observed OTUs (≥ 97% similarity; 349 ± 17 in Bangladeshi children versus 249 ± 10 in U.S. children; P<0.0001) (Figure 1B and Figure S2). Together, these results suggest that factors associated with habitation within distinct geographical regions may strongly influence the diversity of the gut microbiota in childhood.

### Bangladeshi and U.S. distal gut microbiota are compositionally distinct

**Figure 2:**
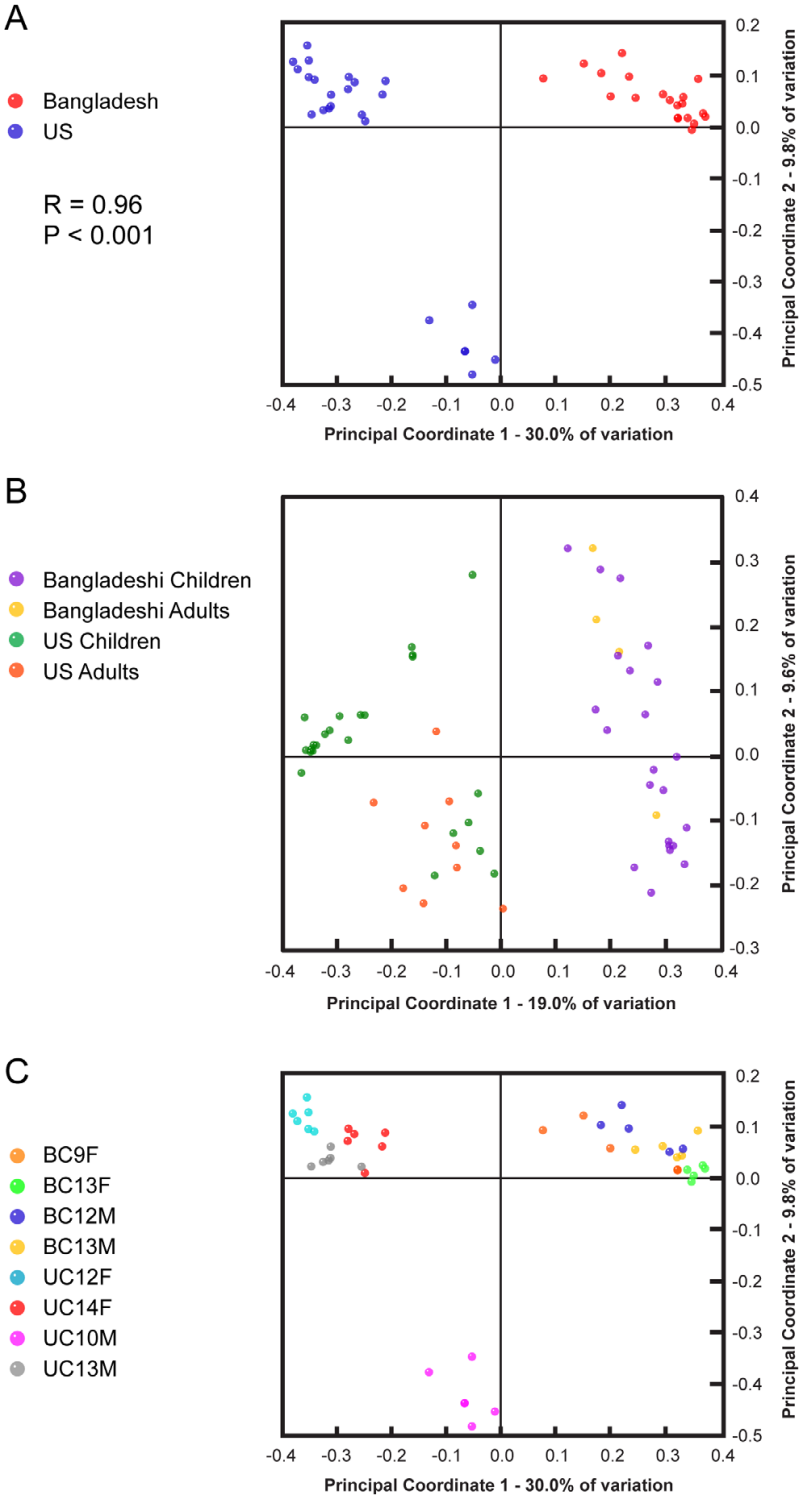
Principal Coordinates Analysis of unweighted UniFrac distances for 16S rDNA V1–V3 sequence data. Beta diversity patterns were explored using Principal Coordinates Analysis (PCoA). Samples were rarefied to 6,596 reads/sample for Figs. 2A and 2C and rarefied to 427 reads/sample for 2B due to the smaller number of available reads per U.S. adult. The near full-length 16S rDNA data were not included in this analysis. (A) The sample symbols for Bangladeshi and U.S. children are colored by country. Only the data from the children (with the exception of BK8F and BK9F) were included in this PCoA plot. (B) Nine U.S. adults were included in this analysis (one time point per adult). The fecal communities for all three subjects (Subjects A, B, and C) in the Eckburg *et al.* study (near full-length 16S rDNA sequences) and all 6 subjects (Subjects A, B, C, D, E, F) in the Dethlefsen *et al.* studies (V1–V3 16S rDNA sequences) were included in the analysis [11], [25], [26]. The near full-length 16S rDNA sequences were filtered *in silico* to the V1–V3 regions using ARB. Data from all of the children were included in this analysis. (C) Same as (A) but with each sample symbol colored-coded by subject.

In our study, phylogenetic composition-based sample clustering was associated with subject country of residence. In unweighted UniFrac PCoA, the first principal coordinate (PC1) captured 30% of intersample variance and revealed a sharp distinction between the gut microbiota of Bangladeshi and U.S. children (Figure 2A). This difference was significant with a non-parametric permutation analysis of similarity (ANOSIM) test, analogous to the univariate ANOVA (analysis of variance) test, where R = 0.96 (p<0.001). Parallel results were obtained with weighted UniFrac analysis, where PC1 (55%) and PC2 (14%) resolved differences between Bangladeshi and U.S. children (Figure S3A), and with Bray-Curtis analysis (Figure S3B). When nine U.S. adult samples from three previously published studies [11], [25], [26] were included in the unweighted and weighted UniFrac analyses, the country clusters remained distinct (Figure S3C). Distinct clusters also distinguished U.S. adults and Bangladeshi adults (R = 0.65; p<0.001), and U.S. adults and Bangladeshi children (R = 0.76; p<0.001; Figure 2B and Table S1A).

**Figure 3:**
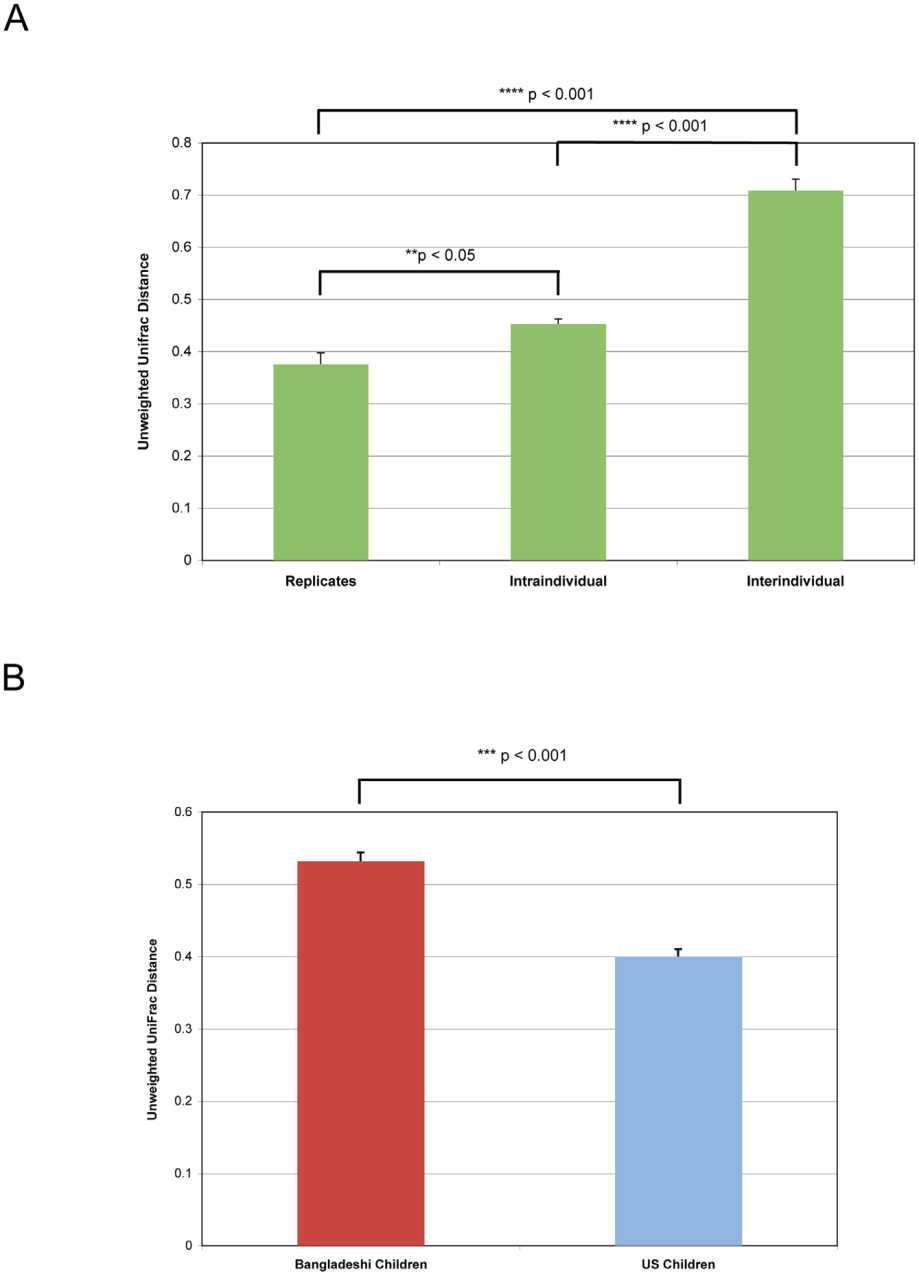
Mean pairwise unweighted UniFrac distance for subsets of samples. Mean pairwise unweighted UniFrac distance reflecting (A) sample-to-sample variation among replicates (left), within individuals over time (center), and between individuals (right), and (B) the average month-to-month intraindividual variation (distances between samples spanning one month) within Bangladeshi children (left) and within U.S. children (right). In A, the replicates from all six Bangladeshi adults and U.S. child 10 M were included in the analysis (Table 1). In all others (A and B), UniFrac distance calculations excluded the two additional Bangladeshi children (BK) and the Bangladeshi adult (BA) subjects because only one sample was collected from each of these individuals (Table 1). Means are shown ± SEM, and p-values were calculated by a two-tailed t-test with unequal variance.

Looking beyond geography, PC2 (9.8% of variance) and PC3 (7.8% of variance) separated samples based on individual subject, and varying levels of intra-individual clustering of monthly time points occurred in all children (Figure 2C and Figure S4). Similar unweighted UniFrac PCoA findings were attained at different OTU thresholds (90%, 95%, and 99%) (data not shown). All children were distinct from each other (ANOSIM pairwise comparisons generated an R>0.5; p<0.05) except Bangladeshi child 9F when compared to Bangladeshi child 12 M or Bangladeshi child 13 M (Table S1B). The observation that the three Chinese-American children clustered separately from the only European-American child in the cohort may be due to dietary or genetic factors. Clustering associated with gender (R = 0.07; p<0.05), age, seasonality, collection month, nutrition status, and mother's education level was not observed, although the number of individuals in this study was small and may have precluded the identification of significant differences based on these variables (Figures S5A and S5B; additional data not shown). Averaged, unweighted UniFrac distances supported observations from previous studies indicating that inter-individual variation is greater than intra-individual variation (Figure 3A) [38]. Unweighted and weighted UniFrac analysis revealed a tight clustering pattern between pairs of biological or technical replicates (Figures S4 and S5C). The variation between replicate pairs (measured by unweighted UniFrac distances) was significantly less than intra- or inter-individual variation (Figure 3A). Taken together, these results suggest the possibility that the gut microbiota of children in Bangladesh and the United States is distinct, and that children in these two locales show both compositional individuality and temporal stability in their distal gut microbiota. Further studies and additional subjects are necessary to confirm these results.

### Taxonomic differences between microbiota of U.S. and Bangladeshi children

**Figure 4:**
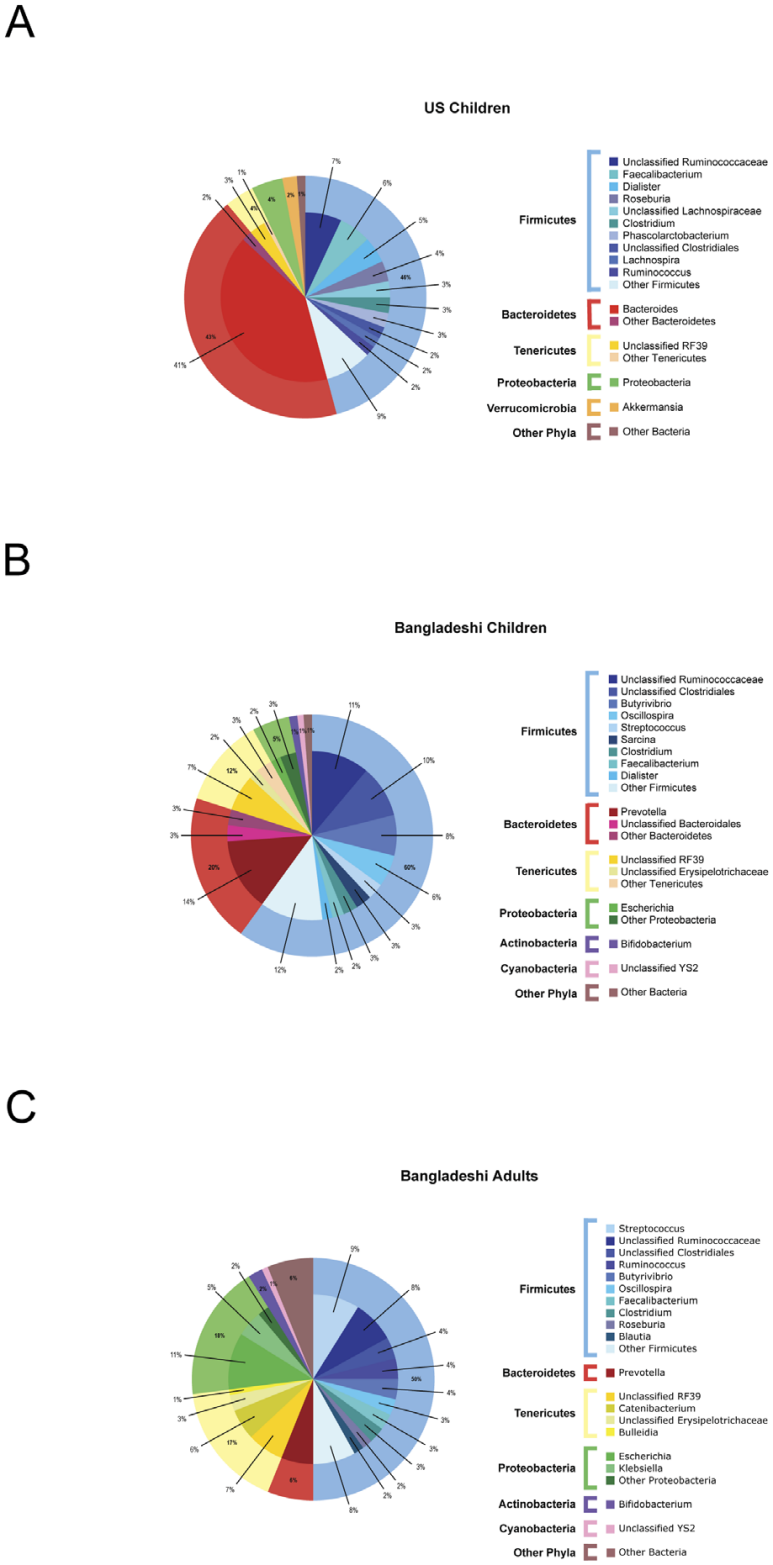
Mean relative abundance of bacterial taxa (≥1%) in the V1–V3 16S rDNA sequence data. The composition of the distal gut microbiota of (A) U.S. children (B) Bangladeshi children, and (C) Bangladeshi adults is displayed. The outer ring reflects the mean relative abundance of the most abundant phyla (e.g., Firmicutes, blue; Bacteroidetes, red) and the inner ring represents the most abundant genera (with a gradient of shading corresponding to the phylum colors). Only U.S. children, Bangladeshi children, and Bangladeshi adult subjects were included in this analysis.

The vast majority (99.1%) of V1–V3 sequences were assigned to six dominant phyla previously found in the human distal gut: Firmicutes (50.6%), Bacteroidetes (30.0%), Proteobacteria (8.7%), Tenericutes (7.9%), Verrucomicrobia (1.1%), and Actinobacteria (0.9%). A PCoA phylum biplot indicated that Firmicutes were more prevalent in the Bangladeshi children than in the U.S. children and that the opposite was true for Bacteroidetes (Figure S6A). Firmicutes (46%) and Bacteroidetes (43%) evenly dominated the gut microbiota of the U.S. children with modest representation of Tenericutes (4%), Proteobacteria (4%) and Verrucomicrobia (2%) (Figure 4A). In contrast, Firmicutes outnumbered Bacteroidetes 60% to 20% in the Bangladeshi children with a similar relative abundance of Proteobacteria (5%) as in the U.S. children and a greater amount of Tenericutes (12%) (Figure 4B). Some of these features were also observed in the Bangladeshi adults, namely, a high relative abundance of Firmicutes (50%) and a low abundance of Bacteroidetes (6%) (Figure 4C).

At the genus level, more pronounced differences were observed between the Bangladeshi and U.S. children. Within the phylum Bacteroidetes, the genus *Prevotella* was the most prevalent genus in Bangladeshi children and adults, while the *Bacteroides* genus was the most prevalent in U.S. children (Figure 4 and Figure S6B). After *Bacteroides* (41%), the most abundant taxa in U.S. children included the family Ruminococcaceae (7%) and the genera *Faecalibacterium* (6%), *Dialister* (5%), and *Roseburia* (4%) within the phylum Firmicutes (Figure 4A). U.S. and Bangladeshi children were both colonized by *Faecalibacterium prausnitzii*, a prominent butyrate-producer. *Dialister invisus*, a Gram-negative, obligate anaerobe was highly abundant in U.S. child 10 M (Figure S6B). All of the Verrucomicrobia-associated reads found in the U.S. children were classified as *Akkermansia muciniphila* (2%) (Figure 4A). U.S. children 10 M and 13 M were particularly enriched in this species. After *Prevotella* (14%), the most prominent taxa in the Bangladeshi children included the family Ruminococcaceae (11%), the order Clostridiales (10%), and the genera *Butyrivibrio* (8%) and *Oscillospira* (6%) of the Firmicutes phylum (Figure 4B). Other relatively abundant taxa in Bangladeshi children included the putative order RF39 within Tenericutes (7%), the genus *Bifidobacterium* within Actinobacteria, and the lineage YS2 related to Cyanobacteria (1%). As with the U.S. and Bangladeshi children, the Ruminococcaceae family (8%) and the putative order RF39 (7%) were prevalent in Bangladeshi adults (Figure 4C). Like Bangladeshi children, Bangladeshi adults also featured the putative order YS2 (1%), the order Clostridiales (4%), the genus *Bifidobacterium* (2%), and the genus *Butyrivibrio* (4%).

**Figure 5:**
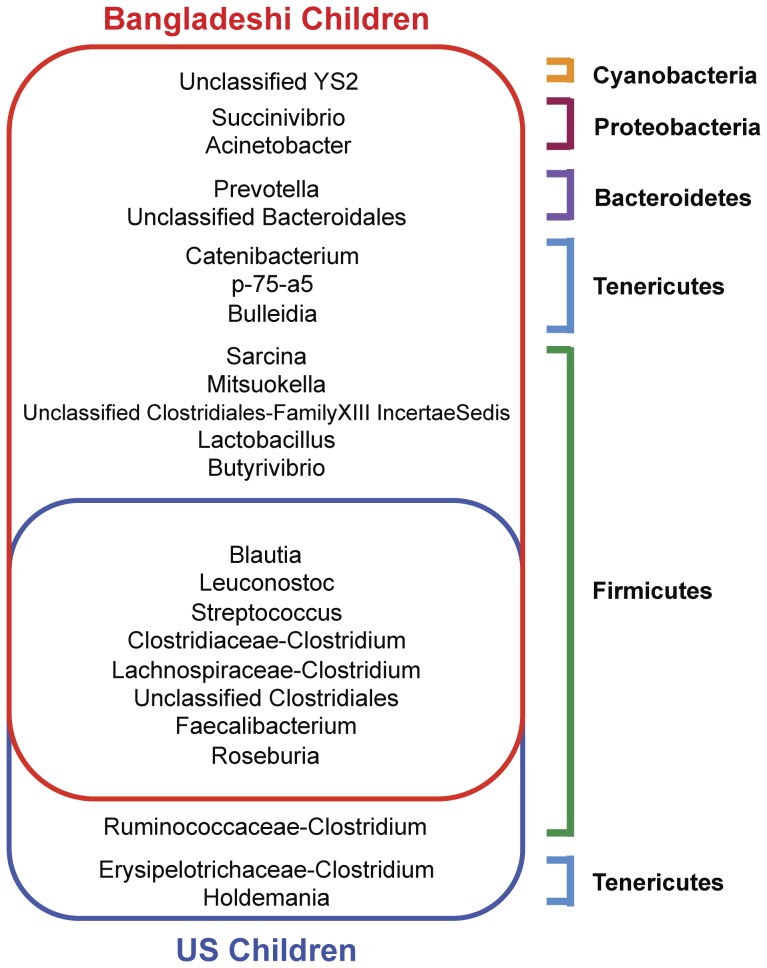
Venn diagram of the exclusive and shared genera of Bangladeshi and U.S. children based on V1–V3 16S rDNA sequence data. The G test of independence was used to determine whether genus presence or absence was associated with one group of children or the other. A list of the most significant genera was compiled based on FDR-corrected p-values. Furthermore, to qualify for a specific country group, the genus had to be observed ≥5 times for each time point within an individual and within all individuals in a country group. The genera exclusively found in Bangladeshi or U.S. children are outlined in red and blue, respectively. The overlapping area between the two groups represents the genera that are shared by all individuals from both countries. The size of each enclosed area is directly proportional to the number of genera that are found in each group. The genera are colored-coded according to the list of phyla on the right. Only U.S. children and Bangladeshi children were included in this analysis.

**Figure 6:**
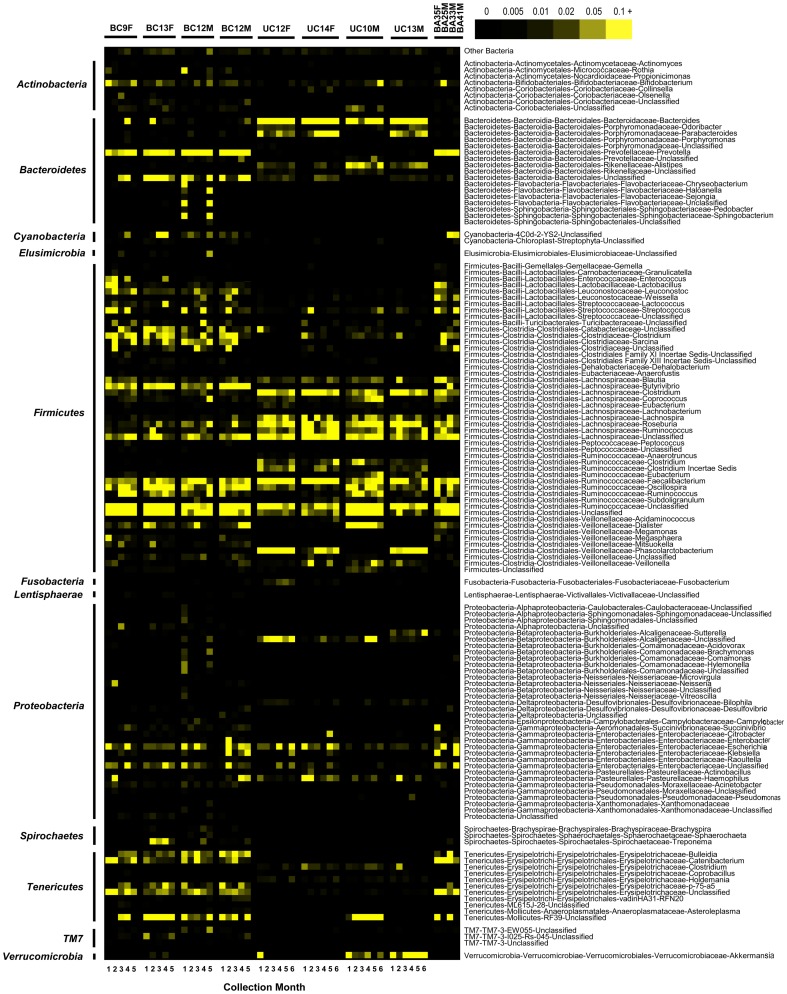
Heat map of the relative abundance of genera found in the V1–V3 16S rDNA sequence data. Data from U.S children, Bangladeshi children, and Bangladeshi adults were rarefied to the same number of reads per sample to normalize for the unequal sampling effort. Only genera with a total normalized abundance of at least ten reads are displayed. Color intensity is proportional to the relative abundance of the taxon and is represented by the scale (black, 0% present; yellow, ≥10% present).

Eight taxa were shared between Bangladeshi and U.S. children, three taxa were found only in the U.S. children, and thirteen taxa were exclusive to Bangladeshi children (Figure 5). The taxa shared by Bangladeshi and U.S. children consisted of *Blautia*, *Leuconostoc*, *Streptococcus*, Clostridiaceae-associated *Clostridium*, Lachnospiraceae-associated *Clostridium* (here and below, such lineages contain sequences misidentified as *Clostridium* and lack other named sequences), unclassified Clostridiales, *Faecalibacterium*, and *Roseburia* (Figure 5). The three taxa that were found only in the U.S. children included Ruminococcaceae-associated *Clostridium*, Erysipelotrichaceae-associated *Clostridium*, and *Holdemania*. The taxa exclusive to the Bangladeshi children were affiliated with the phyla Bacteroidetes (*Prevotella*, unclassified Bacteroidales), Firmicutes (*Sarcina*, *Mitsuokella*, unclassified Clostridiales-FamilyXIII IncertaeSedis, *Lactobacillus*, *Butyrivibrio*), Tenericutes (*Catenibacterium*, *p-75-a5*, *Bulleidia*), Proteobacteria (*Succinivibrio*, *Acinetobacter*), and Cyanobacteria (unclassified YS2) (Figure 5). The most abundant *Lactobacillus* species present in the Bangladeshi children were *L. salivarius*, *L. ruminis*, *L. gasseri*, and *L. crispatus*. Besides the *Lactobacillus* genus, *Butyrivibrio* was also identified as being exclusive to Bangladeshi children. Of the thirteen taxa found in Bangladeshi children, but not U.S. children, *Prevotella*, *Lactobacillus*, *Butyrivibrio*, *Catenibacterium*, and unclassified YS2 were also present in Bangladeshi adults (Figures 4C, 5, and 6).

**Figure 7:**
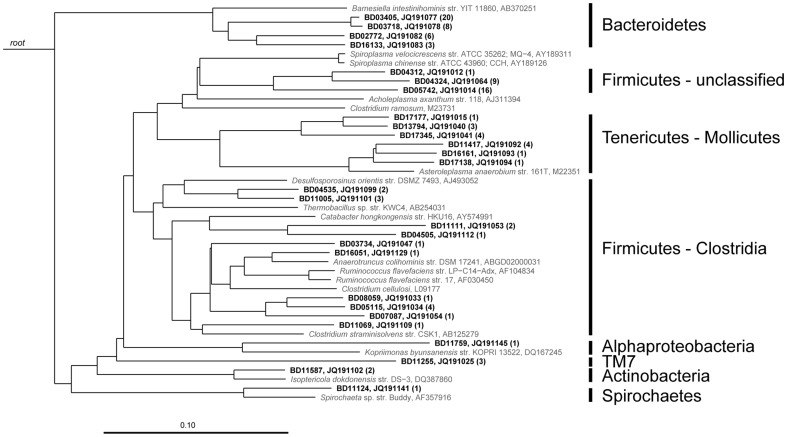
Phylogeny of the most novel OTUs found in the distal gut microbiota of children from Bangladesh. Neighbor joining tree based on near full-length 16S rDNA sequences showing the 27 OTU representatives (in bold) from the Bangladesh dataset that have less than 95% sequence similarity to published sequences, and their Genbank accession numbers. The tree was rooted with the archaeal 16S ribosomal RNA gene from *Halococcus morrhuae* (X00662). The number of clones for each OTU in the near full-length dataset is shown in parentheses. Their closest publicly-available sequences are shown in gray font. The horizontal scale bar indicates 10% sequence divergence.

At the level of OTU, re-sampling-based multiple hypothesis testing was used to identify OTUs that significantly differed in relative abundance between the Bangladeshi and U.S. children (Table 3). The two OTUs with relative abundances that were significantly higher in U.S. children than in Bangladeshi children were *Bacteroides thetaiotaomicron* and *Faecalibacterium prausnitzii*. The two OTUs that were significantly more abundant in the Bangladeshi children were classified within *Butyrivibrio* and Clostridiales (Table 3). That these two OTUs could not be resolved to a finer level of taxonomy (unassigned by the Greengenes database) is indicative of the presence of novel or unclassified species in the Bangladeshi children. This point is further underscored by the observation that 27 of the 185 novel OTUs from the set of near full-length sequences, shared less than 95% sequence similarity with any publicly-available sequence (Table 4 and Figure 7). The availability of near full-length sequences enabled species-level classification of a small proportion of the *Prevotella* OTUs found in the Bangladeshi children (data not shown). Only 216 of the 947 near full-length *Prevotella* sequences were classifiable at the species level (at a 99% similarity threshold) to a publicly-available, named sequence. Of these 216 classified, near full-length *Prevotella* sequences, 90% matched *Prevotella copri* and 7% matched *Prevotella stercorea*, two types of Gram-negative, anaerobic rods [39]. Representing 2% of the 216 sequences, *Prevotella ruminocola* has a predominant role in digesting hemicelluloses and pectin [40]. Overall, these results demonstrated that features shared with Bangladeshi adults – a preponderance of *Prevotella* and *Butyrivibrio*, and a lack of *Bacteroides*, among other taxa – contributed heavily to the compositional differences between Bangladeshi child distal gut microbiota and the microbiota of U.S. children.

**Table 3:** OTUs whose relative abundance differed significantly between U.S. and Bangladeshi children.

OTU ID	Phylum	Lowest Assignable Taxonomic Level	Relative Abundance		AdjustedP-value
			BangladeshiChildren	U.S. Children	
39957	Firmicutes	Clostridiales (O)	6.58×10^−4^	7.06×10^−5^	0.0210
37039	Firmicutes	*Butyrivibrio* (G)	2.16×10^−2^	3.21×10^−6^	0.0210
27010	Bacteroidetes	*Bacteroides thetaiotaomicron* (S)	<1.00×10^−6^	6.29×10^−4^	0.0210
41194	Firmicutes	*Faecalibacterium prausnitzii* (S)	6.19×10^−5^	1.08×10^−2^	0.0738

The OTU table was filtered to retain the 836 most abundant OTUs to avoid over-adjusting for excessive multiple tests. A re-sampling-based multiple testing procedure was used to test the null hypothesis of no significantly different OTU relative abundances between Bangladeshi and U.S. children. Each significantly different OTU was assigned to the lowest taxonomic level (O = order, G = genus, S = species). The relative abundance for Bangladeshi and U.S. children is listed for each OTU. Permutation adjusted p-values for this step-down multiple testing procedure were calculated [60]. Since these p-values were radically adjusted, a p = 0.10 threshold of significance was used.

**Table 4:** Novel OTUs found in the gut microbiota of children from Bangladesh.

Phylum	Lowest Assignable Taxonomic Level	OTU Representative	GenBank Accession Number	Number of Clones	Sequence Identity (%)*	Closest Published Neighbor	Environment
Firmicutes	Lachnospiraceae (F)	16051	JQ191129	1	92.27	AM277638	human stool
Firmicutes	Clostridiales (O)	11069	JQ191109	1	93.08	EU464209	giraffe stool
Firmicutes	Clostridiales (O)	05115	JQ191034	4	91.75	GQ449178	cow stool
Firmicutes	Clostridiales (O)	08059	JQ191033	1	92.87	FJ508143	human gut biopsy
Firmicutes	Clostridiales (O)	07087	JQ191054	1	94.27	EU464146	giraffe stool
Firmicutes	Clostridiales (O)	03734	JQ191047	1	94.39	AB606357	mouse stool
Firmicutes	Clostridiales (O)	04505	JQ191112	1	91.96	GQ448104	cow stool
Firmicutes	Clostridiales (O)	11111	JQ191053	2	92.10	GU303975	cow rumen
Firmicutes	Clostridiales (O)	04535	JQ191099	2	94.91	JF163688	human skin
Firmicutes	Clostridiales (O)	11005	JQ191101	3	94.72	EU887982	leach bed reactor
Tenericutes		04324	JQ191064	9	92.84	JF217071	human skin
Tenericutes		04312	JQ191012	1	94.89	DQ325546	human stool
Tenericutes		05742	JQ191014	16	93.98	AF132232	human stool
Tenericutes	*Asteroleplasma* (G)	11417	JQ191092	4	91.22	EF593052	predigester slurry of biogas plant
Tenericutes	*Asteroleplasma* (G)	16161	JQ191093	1	90.40	EF593052	predigester slurry of biogas plant
Tenericutes	*Asteroleplasma* (G)	17138	JQ191094	1	91.00	NR044657	predigester slurry of biogas plant
Tenericutes	Mollicutes (O)	13794	JQ191040	3	90.65	EF445265	cow rumen
Tenericutes	Mollicutes (O)	17177	JQ191015	1	90.64	EU473447	Somali wild ass stool
Tenericutes	Mollicutes (O)	17345	JQ191041	4	91.80	FJ685412	cow stool
Actinobacteria	*Brachybacterium* (G)	11587	JQ191102	2	94.32	FN563163	biogas reactor
Proteobacteria	Alphaproteobacteria (C)	11759	JQ191145	1	91.04	AB299553	termite gut
Bacteroidetes	Porphyromonadaceae (F)	03405	JQ191077	25	91.38	EU452376	mouse skin
Bacteroidetes		03718	JQ191078	8	91.32	HM832482	mouse skin
Bacteroidetes	Bacteroidales (O)	02772	JQ191082	6	91.88	EU464528	elephant stool
Bacteroidetes		16133	JQ191084	3	89.80	EU461142	Hamadryas baboon feces
TM7	TM7-3 (O)	11255	JQ191025	3	94.33	FJ671754	cow feedlot
Spirochaetes	*Spirochaeta* (G)	11124	JQ191141	1	92.86	DQ353914	gorilla stool

* Sequence identity to closest published neighbor longer than 1000 base pairs.

The following near full-length 16S rDNA sequences shared less than 95% similarity to sequences available in GenBank. Each novel OTU was assigned to the lowest taxonomic level (O = order, F = family, G = genus). Five OTUs could not be resolved past the phylum level. The following features are listed for each novel OTU: phylum, lowest level taxonomic classification, OTU representative ID, GenBank accession number, absolute abundance of clones (out of 8,039 total), percent sequence similarity shared with the closest published neighbor, GenBank accession number of closest published neighbor, and environment where closest published neighbor was discovered.

### Gut microbiota composition is less stable over time in Bangladeshi versus U.S. children

Overall, the U.S. children exhibited a higher degree of intra-individual similarity over time, as measured by unweighted UniFrac distance, compared to the Bangladeshi children (0.40 versus 0.53 respectively; p<0.001; Figure 3B). Intrapersonal monthly variation correlated inversely with age in the Bangladeshi child cohort, although the small number of children in this study prevents a robust analysis of this issue (Figure S7A). This age-related correlation was not observed among the U.S. child cohort (p>0.05). At the phylum level, a greater degree of variation in relative abundance was observed between the consecutive monthly samples from the Bangladeshi children compared to the U.S. children (Figure S8). Comparing the average unweighted UniFrac distance for only consecutive time points (rather than all time points, as above) between the two groups underscores the greater intra-individual temporal variation in the Bangladeshi children relative to the U.S. children (0.52 versus 0.39 respectively; p<0.001) (Figure S7B). Temporal autocorrelation was modeled to ensure that the time interval between samplings did not bias the unweighted UniFrac distances (Figure S7C).

Temporal variability was also observed at the genus level within both the U.S. and Bangladeshi children (Figure 6), especially among the most abundant genera. Three of the four U.S. children (U.S. children 12F, 14F, and 13 M) experienced more than 10% change in the relative abundance of their *Bacteroides* population for three out of five monthly transitions (Figure S9A). The most dramatic shift in the *Bacteroides* population occurred between the second and third months for U.S. child 14F, when the population sharply increased from 24% to 69% (a change of 45%). Large fluctuations in the relative abundance of *Prevotella* were observed in two of the Bangladeshi children, 12 M and 13 M (Figure S9B). Interestingly, Bangladeshi child 12 M, the only stunted child in the cohort (height-for-age Z-score<−2.0), experienced the highest *Prevotella* fluctuations from month to month with absolute differences ranging from 42% to 57%.

### Near full-length 16S rRNA sequences and novel OTUs

The 8,039 near full-length sequences generated from 15 Bangladeshi samples (five monthly samples from Bangladeshi child 13F, Bangladeshi child 12 M, and Bangladeshi child 13 M; Table 1) were clustered into 503 OTUs by manual alignment and 536 OTUs using mothur, with a Chao 1 estimated richness of 760 OTUs (Figure S10A). Overall, the near full-length data closely matched the pyrosequencing data (Figure S10B). The average pairwise weighted UniFrac distance between near full-length and V1–V3 data from the same sample (0.16 ± 0.01) was less than the average distance between either V1–V3 or near full-length data from different samples of the same individual (0.37 ± 0.02 versus 0.38 ± 0.01 respectively; Figure S10C). Although the near full-length and V1–V3 data from Bangladeshi child sample 12M4 appeared to differ significantly, the average UniFrac distance (0.30) for these within-sample comparisons was less than the average intraindividual variation (Figure S10C).

Of the 503 OTUs from these Bangladeshi children, 185 (36.8%) were considered novel using a 99% threshold for the manually-aligned and masked near full-length sequences. Twenty-seven novel OTUs shared less than 95% sequence similarity with any publicly-available sequence (Table 4 and Figure 7). Clone 03405 of the family Porphyromonadaceae (phylum Bacteroidetes) was the most highly represented novel sequence (25 observations) and had 91.38% similarity to HM832482 (recovered from mouse skin [41]). The OTUs with the least similarity to any publicly-available sequence included clone 16133 (phylum Bacteroidetes) with 89.80% similarity to EU461142 (recovered from hamadryas baboon feces [42]), clone 16161 (genus *Asteroleplasma*) with 90.40% similarity to EF593052 (recovered from the predigester slurry of a biogas plant; unpublished data), clone 17177 (order Mollicutes) with 90.64% similarity to EU473447 (recovered from Somali wild ass stool [42]), and clone 13794 (order Mollicutes) with 90.65% similarity to EF445265 (recovered from cow rumen [43]).

## Discussion

Overall, we found that Bangladeshi children harbor greater bacterial diversity and evenness in the distal gut than U.S. children. Our results are similar to previous observations of higher bacterial diversity in children living in rural Burkina Faso compared to children from urban Italy [6]. Our findings also align with those of a 2012 study revealing that Venezuelan Amazonas and rural Malawian adults had higher bacterial diversity compared to U.S. adults living in metropolitan areas [7]. Notably, a 2011 study demonstrated that healthy Bangladeshi children (ages 2–3 years) had higher microbial diversity relative to malnourished children [5]. As an aside, we found that inter-individual variation in the fecal microbiome of these subjects is greater than intra-individual variation, a finding that is supported by a Human Microbiome Project study involving the largest cohort to date (242 individuals) [38].

In our study, the differences in the fecal microbiomes of these Bangladeshi and U.S. children reflect a number of potentially important, and interwoven contributing factors, such as socioeconomic status, genetics, dietary habits, sanitary, and other environmental conditions. In an effort to focus exclusively on variation in the distal gut microbiome during states of health, we carefully selected the four Bangladeshi children for our study (9F, 13F, 12 M, and 13 M) according to stringent and consistent criteria, including an absence of diarrhea or confirmed enteric infectious disease for 8 consecutive months in total. Of the existing ICDDR,B cohort of ∼420 children, only six children (including the four we studied) met these criteria. It is possible that our selection of children based on the absence of recent diarrhea and enteric infections is associated with the observed high diversity of their distal gut microbiota; diversity is thought to confer resilience by ensuring functional redundancy and ecosystem stability [44], [45], [46]. This explanation is supported by a 2012 study in which a cohort of healthy Bangladeshi children exhibited higher bacterial diversity compared to their siblings recovering from acute diarrheal illness [47]. Further studies involving more subjects are necessary to understand how the gut microbial diversity of Bangladeshi children with recent intestinal illness would compare to that of healthy children in Bangladesh or the U.S.

The patterns of diversity in this small subset of Bangladeshi children may be either the result or the cause of the recent absence of diarrheal illness (or be unrelated). With respect to the possibility of a contributing or causal role, the diverse microbiota of these Bangladeshi children may decrease susceptibility to pathogen invasion by preventing access of pathogens to the appropriate target cells or tissues [48], competing for nutrients [49], secreting antimicrobial peptides [50], and/or modulating the immune system [51]. Compared to conventional mice, strains of mice with low gut microbiota diversity are more susceptible to *Salmonella enterica* colonization and enterocolitis [52]. Reduced gut microbiota diversity has been implicated in several morbidities such as inflammatory bowel diseases [53] and diarrhea [54]. In our study, *Bifidobacterium* was enriched and *Lactobacillus* was exclusively found in Bangladeshi children. Numerous studies have investigated the properties of specific strains of lactobacilli and bifidobacteria that prevent pathogen invasion through competitive exclusion, production of antimicrobials, and other mechanisms [55]. Stecher *et al.* hypothesize that closely-related species may increase the likelihood of gut colonization by newly-incoming species due to favorable conditions that maximize growth for both the resident and incoming species [52]. This concept could potentially enable a more targeted use of probiotics to prevent gastrointestinal infections in Bangladeshi children who live in slum settings.

Dietary factors may be particularly important in explaining the observed differences in the composition of the distal gut microbiota between the Bangladeshi and U.S. children. The diet of the Bangladeshi children was carbohydrate-rich and heavily dependent on rice, bread, and lentils with rarely any meat. Their U.S. counterparts consumed typical Western diets including animal fat and protein in addition to carbohydrates and vegetables. In a 2009 study of U.S. adult twins (ages 21–32 years) consuming typical Western diets, obese individuals exhibited a higher Firmicutes to Bacteroidetes ratio than lean individuals [56]. Therefore, based on weight assumptions (weight data were not collected in our U.S. cohort), one would predict that the Firmicutes to Bacteroidetes ratio in the U.S. children would be higher than the ratio in the Bangladeshi children. However, in our study, the Firmicutes to Bacteroidetes ratio was 1∶1 in the U.S. children, and the ratio was 3∶1 in the Bangladeshi children. Perhaps this departure from expected results is attributable to age or geographical differences. Another possibility is that our results support the idea that the Firmicutes to Bacteroidetes ratio varies widely across healthy individuals, as was observed in the recent study of the gut microbiota from 242 U.S. adults (ages 18–40 years) [38]. A larger survey of Bangladeshi and U.S. children is necessary to understand the proportions of Firmicutes and Bacteroidetes in relation to their metabolic pathways. Within the Bacteroidetes phylum, *Prevotella* dominated the gut microbiota of the Bangladeshi children while *Bacteroides* dominated the U.S. microbiota. These results show striking similarities to the findings of a 2010 study comparing the fecal microbiota of children from Burkina Faso who consume diets high in carbohydrates and low in protein with that of children from Italy who consume typical Western diets rich in meat, dairy, and carbohydrates. *Prevotella* were highly abundant in the microbiota of the children living in Burkina Faso, in contrast to the children in Italy whose microbiota was dominated by *Bacteroides* [6]. A *Bacteroides*-dominated gut community is significantly associated with long-term diets rich in animal protein and saturated fat, while a *Prevotella*-dominated community is associated with a plant-based diet high in carbohydrates and lacking in meat and dairy products [57]. *Parabacteroides* (present in U.S. children 12F, 14F, and 13 M), *Alistipes* (present in all four U.S. children), and other taxa that co-occur with a *Bacteroides*–dominated signature (Figure 6) are particularly adept at fermenting carbohydrates and protein to extract energy [57], [58]. Conversely, *Catenibacterium* (present in all 4 Bangladeshi children) and other bacteria that co-occur with a *Prevotella*-dominated gut community (Figure 6) degrade mucin and simple sugars [57], [58]. Although our study examined only a small number of subjects, our results support an association between diet and gut microbiota. Our findings also underscore the importance of characterizing gut microbiota composition in subjects residing in varied geographical contexts [6], [7]. For example, features of diet are shared between residents of Burkina Faso and Bangladesh, whereas other factors (confounded with diet in comparisons to Western populations) such as host genetics, rural versus urban, are not.

To our knowledge, this study is the first to compare the temporal stability of distal gut microbiota of primary school-age children and adolescents in developing and developed countries. To date, most previous studies have focused on the temporal variation of the microbiota of infants and adults from developed countries. In these previous studies, dramatic perturbations in the gut microbiota observed over multiple time points have corresponded with significant changes in diet or antibiotic usage [11], [26], [59]. In our study, consecutive monthly samples from the Bangladeshi children exhibited a greater degree of variation in the relative abundance of phyla compared to the monthly samples from the U.S. children. Since the Bangladeshi and U.S. children were closely monitored and did not receive antimicrobial therapy for 8 or 9 consecutive months, the higher temporal variation observed in the Bangladeshi children is likely not attributed to antibiotic usage. Although diet was not controlled for in either group, in general, Bangladeshi children from low-income families have access to a smaller variety of foods compared to U.S. children from affluent families. Hence, variability in the diet probably does not contribute to the larger degree of temporal variation observed in the gut microbiota of Bangladeshi children. One potential, alternative explanation is that a high level of temporal variation may result from more frequent or intensive exposure to bacteria in the environment due to unhygienic conditions, resulting in subclinical perturbations to their gut microbiota. Additional data are needed to test this hypothesis.

Although our key observations align with those of recent studies, due to the small sample size and overall design of this study, we view these results as supportive but not definitive evidence for our conclusions. Recruiting subjects and collecting multiple samples from each individual in the setting of this study is a difficult process, but subsequent studies should control for socioeconomic, genetic, and dietary variables. To begin to understand the role of the gut microbiota in relation to these and other factors, adequately-powered studies directly comparing the gut microbiota of different groups of subjects differing by only one or two of these variables are warranted. Moreover, a large-scale, randomized controlled trial involving targeted interventions would provide a better platform for unraveling these interactions. Extending these experiments to relevant animal models would help in examining possible causal relationships.

In summary, this study expands our knowledge about the distal gut microbiota of primary school-age children and adolescents in developing and developed countries. Healthy Bangladeshi children exhibit stark differences in community structure, greater gut microbial diversity, and higher monthly baseline variation compared to U.S. children, factors that may demand consideration in studies that seek to understand microbiome associations with childhood disease in developing countries. While it is not possible to pinpoint causal relationships underlying the compositional differences between the microbiota of these Bangladeshi and U.S. children, dietary, genetic, and environmental factors are all likely to play a role. These results may help direct the design of future studies and contribute to a much-needed foundation for the future implementation of strategies for manipulating the microbiota of children to enhance and restore health.

## Supporting Information

Table S1 **A. ANOSIM Pairwise Comparisons By Group.** The non-parametric permutation analysis of similarity (ANOSIM) test was used to calculate the global test statistic R. **B. ANOSIM Pairwise Comparisons By Child.**
(DOCX)

Figure S1 **Rarefaction curves of 16S rDNA V1–V3 sequence data.** (A) The rarefaction curve for the entire V1–V3 sequence dataset. The blue line represents the observed species richness. The red line represents the range of estimated OTU richness as predicted by the Chao1 non-parametric estimator and indicates that the total OTU richness is much higher than currently observed with our V1–V3 pyrosequencing effort. The error bars on the Chao1 estimator correspond to the lower and upper bound 95% confidence intervals. (B) The individual rarefaction curves are color-coded according to the list of subjects on the right. The rarefaction curves for the Bangladeshi children (BC) were generated using all 5 monthly time points per individual, while the rarefaction curves for the US children (UC) were generated using all 6 monthly time points per individual.
(AI) Click here for additional data file.

Figure S2 **Phylogenetic diversity rarefaction curves.** The phylogenetic diversity metric is a branch length-based measurement. Samples were rarefied to an equal number of reads to normalize the data from uneven sampling efforts. In these rarefaction curves, phylogenetic diversity is a function of sequencing effort, and color-coded points correspond to the individuals listed on the right. Only U.S. children (lines) and Bangladeshi children (circles) were included in this analysis.
(AI) Click here for additional data file.

Figure S3 **Principal Coordinates Analysis of UniFrac distances for V1–V3 sequences by country.** Samples were rarefied to an equal number of reads to normalize the data from unequal sampling efforts. The OTU counts were log transformed (base 2), and the (A) weighted UniFrac distances and (B) Bray-Curtis intersample distances (dissimilarities) were calculated and plotted. Unlike unweighted UniFrac, relative abundance is incorporated in weighted UniFrac and Bray-Curtis measurements. (C) Unweighted UniFrac distances are illustrated on this PCoA plot including the 9 adult samples as previously described in Figure 2B (Bangladeshi children, red points; U.S. children, cyan points).
(AI) Click here for additional data file.

Figure S4 **UPGMA of UniFrac distances for V1–V3 sequences.** Unweighted pair group method with arithmetic mean (UPGMA) translates the structure in a pairwise UniFrac distance matrix into a rooted dendrogram. A jackknifing analysis was performed to measure the robustness of the UPGMA results. This process was repeated 100 times. Jackknife values for nodes with less than 0.80 support were not displayed. Samples are color-coded according to the key shown on the left. The horizontal scale bar indicates 4% sequence divergence.
(AI) Click here for additional data file.

Figure S5 **Principal Coordinates Analysis of UniFrac distances for V1–V3 sequences.** PCoA was used to visualize unweighted UniFrac distances and the potential factors influencing gut microbiota. Samples were rarefied to an equal number of reads to normalize the data from uneven sampling efforts. Each Bangladeshi or U.S. child's samples are colored by (A) sex and (B) age. Only U.S. children and Bangladeshi children were included in PCoA plots A and B. (C) Weighted UniFrac was used to illustrate the similarities of technical (all BA and BK samples) and biological replicates (U.S. child 10 M). Details regarding these replicates are available in Table 1 and the Materials and Methods section.
(AI) Click here for additional data file.

Figure S6 **Biplots of unweighted UniFrac distances for V1–V3 sequences.** The biplots display the dominant taxa that explain variation in different areas of the PCoA plot at the (A) phylum or (B) genus level. Country differences are highlighted, and only Bangladeshi children and U.S. children were included in the analysis (Bangladeshi children, red points; U.S. children, cyan red points; phyla, purple circles; genera, green circles).
(AI) Click here for additional data file.

Figure S7 **Unweighted UniFrac distances of V1–V3 sequences.** (A) Unweighted UniFrac distances based on monthly intraindividual variation by subject. P-values were calculated by a two-tailed t-test with unequal variance, and all p-value pairwise comparisons are displayed in the corresponding table. (B) The consecutive monthly intraindividual variation is compared between U.S. and Bangladeshi children. The average distance between month 1 and month 2, month 2 and month 3, and so on, of one subject is compared to the distance between the same consecutive months in another subject. The mean unweighted UniFrac distance is shown ± SEM, and p-values were calculated by a two-tailed t-test with unequal variance. Bangladeshi and U.S. children are in red and blue respectively, and only Bangladeshi children and U.S. children were included in charts A and B. (C) Temporal autocorrelation was modeled and demonstrates that the time interval between samplings did not bias the unweighted UniFrac distances. The slope of the line approaches 0 and the trendline does not fit the data points well (R^2^ = 0.001); therefore, there is virtually no correlation between the number of days between sampling and unweighted UniFrac distances.
(AI) Click here for additional data file.

Figure S8 **Relative abundance at the phylum level.** 16S rDNA V1–V3 sequences were clustered into OTUs and summarized by phyla. These phyla were color-coded according to the scheme on the right. The horizontal axis displays sample collection month and month number: five time points per Bangladeshi child, six time points per U.S. child, and one time point per Bangladeshi adult. “Other Bacteria” includes Elusimicrobia, Lentisphaerae, Thermi, SR1, and Synergistetes.
(AI) Click here for additional data file.

Figure S9 **Relative abundance of dominant genera in Bacteroidetes.** (A) *Bacteroides* is the major genus enriched in the gut communities of U.S. children. The monthly temporal changes in relative abundance of *Bacteroides* are illustrated for all of the U.S. children. (B) In contrast, *Prevotella* is the dominant genus within Bacteroidetes in Bangladeshi children. The monthly temporal changes in relative abundance of the *Prevotella* are illustrated for all of the Bangladeshi children (Bangladeshi child subjects, excluding BK subjects).
(AI) Click here for additional data file.

Figure S10 **Rarefaction curve of near full-length 16S rDNA sequences and weighted UniFrac distance comparisons between near full-length versus V1–V3 sequences.** (A) The rarefaction curve for all near full-length sequences for the 15 time points from subjects Bangladeshi child 12 M, Bangladeshi child 13 M, and Bangladeshi child 13F (Table 1). The blue line represents the observed species richness. The red line represents the range of estimated OTU richness as predicted by the Chao1 non-parametric estimator and indicates that the total OTU richness is much higher than observed with our available near full-length 16S rDNA clone library sampling. The error bars on the Chao1 estimator correspond to the lower and upper bound 95% confidence intervals. (B) Weighted UniFrac PCoA was used to represent the similarity between our near full-length sequencing and V1–V3 pyrosequencing efforts. Near full-length sequences filtered to the V1–V3 region *in silico* were compared to V1–V3 pyrosequencing reads from subjects, Bangladeshi child 12 M, Bangladeshi child 13 M, and Bangladeshi child 13F. (C) The average pairwise weighted UniFrac distance between filtered, near full-length and V1–V3 data from the same sample was quantified. This average distance was compared to the average distance between either V1–V3 or filtered near full-length data from different samples of the same individual. The mean weighted UniFrac distance is shown ± SEM, and p-values were calculated by a two-tailed t-test with unequal variance.
(AI) Click here for additional data file.
