# Correction: Distinct Distal Gut Microbiome Diversity and Composition in Healthy Children from Bangladesh and the United States

**DOI:** 10.1371/journal.pone.0343568

**Published:** 2026-03-02

**Authors:** Audrie Lin, Elisabeth M. Bik, Elizabeth K. Costello, Les Dethlefsen, Rashidul Haque, David A. Relman, Upinder Singh

After this article [1] was published, the following errors were identified in Fig 6. Specifically,

- The second “BC12M” sample (Lanes 15-20) is mislabeled. It should instead be labeled as “BC13M”.
- A discontinuous horizontal line appears between the “Firmicutes-Bacilli-Lactobacillales-Lactobacillaceae-Lactobacillus” and “Firmicutes-Bacilli-Lactobacillales-Enterococcaceae-Enterococcus” rows of lane 20 (BC12M, Month 5).

Corresponding author DAR stated that the discontinuous line is an artifact that arose during production of the heatmap and does not represent data. The original, manually curated genus-level data file associated with the Fig 6 heatmap is provided here as S1 Data. In the corrected version of Fig 6, the two *Lactobacillus* rows in S1 Data were summed prior to visualization, producing a single genus-level row that reflects the total *Lactobacillus* relative abundance (S2 Data). To replicate the visualization approach in Fig 6 in [1] as closely as possible, the data in S2 Data were replotted using the classic TreeView software (Eisen Lab, version 1.60). An updated version of Fig 6 generated directly from S2 Data is provided below. DAR stated that the corrected Fig 6 exhibits the same overall trends and patterns as the original Fig 6 in [1].

The original underlying sequence data to support all results in [1] remain available from the NCBI Short Read Archive under accession SRA057705 (https://www.ncbi.nlm.nih.gov/sra/?term=SRA057705). The Supporting Information files published with [1] continue to be available at Figshare (https://figshare.com/articles/dataset/Distinct_Distal_Gut_Microbiome_Diversity_and_Composition_in_Healthy_Children_from_Bangladesh_and_the_United_States__/154033). The corrected Fig 6 and both the original and corrected data that are the basis for Fig 6 (S1 and S2 Data) are also available at the Stanford Digital Repository (https://purl.stanford.edu/vz702jd4938).

**Fig 6:**
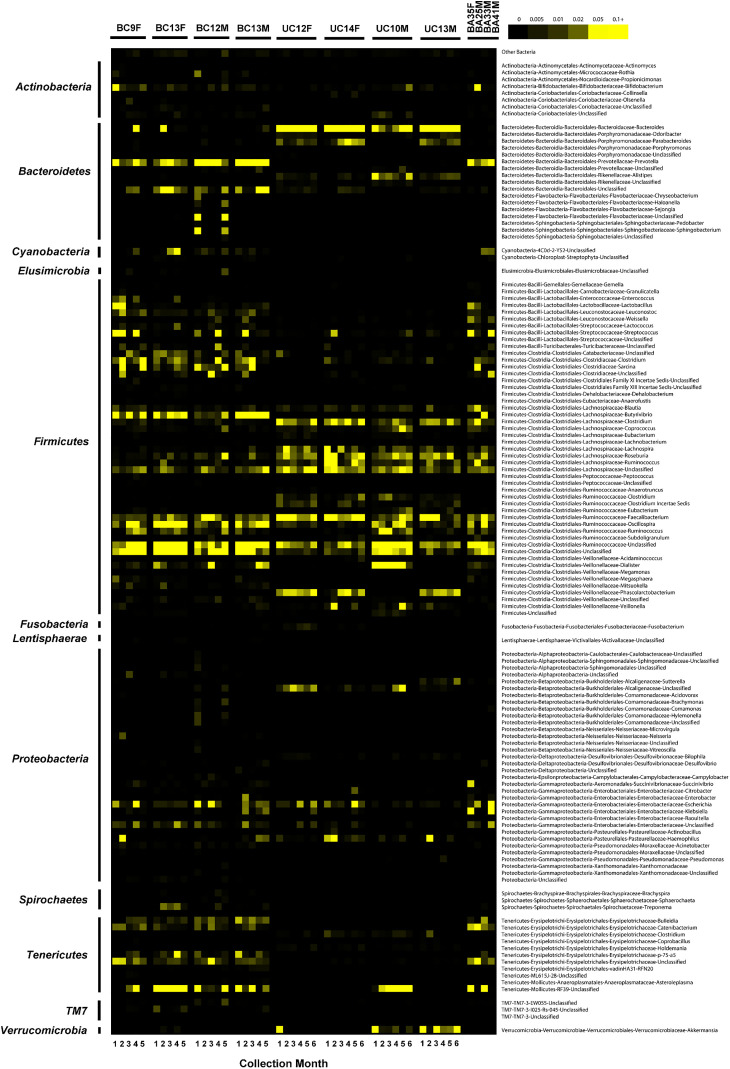
Heat map of the relative abundance of genera found in the V1–V3 16S rDNA sequence data. Data from U.S children, Bangladeshi children, and Bangladeshi adults were rarefied to the same number of reads per sample to normalize for the unequal sampling effort. Only genera with a total normalized abundance of at least ten reads are displayed. Color intensity is proportional to the relative abundance of the taxon and is represented by the scale (black, 0% present; yellow, ≥ 10% present).

## Supporting information

S1 Data Original data underlying the heatmap presented in the uncorrected Fig 6. (XLS)

S2 Data Original data underlying the corrected heatmap in the updated Fig 6, showing the combined *Lactobacillus* relative abundance. (XLS)
