# Supplementary material for: Distinct Distal Gut Microbiome Diversity and Composition in Healthy Children from Bangladesh and the United States
Source: PLoS One. 2013 Jan 22;8(1):e53838. doi: 10.1371/journal.pone.0053838 (PMC3551965; doi:10.1371/journal.pone.0053838)
Supplement: Table S1 — A. ANOSIM Pairwise Comparisons By Group. The non-parametric permutation analysis of similarity (ANOSIM) test was used to calculate the global test statistic R. B. ANOSIM Pairwise Comparisons By Child. (DOCX) [file pone.0053838.s001.docx]

**Table S1 A. ANOSIM Pairwise Comparisons By Group.** The non-parametric permutation analysis of similarity (ANOSIM) test was used to calculate the global test statistic R.

|  | **Bangladeshi Child** | **Bangladeshi Adult** | **U.S. Child** | **U.S. Adult** |
| --- | --- | --- | --- | --- |
| **Bangladeshi Child** |  |  |  |  |
| **Bangladeshi Adult** | 0.12 NS |  |  |  |
| **U.S. Child** | 0.83 *** | 0.79 *** |  |  |
| **U.S. Adult** | 0.76 *** | 0.65 *** | 0.37 *** |  |

*** p < 0.001 ; NS p > 0.05

**Table S1 B. ANOSIM Pairwise Comparisons By Child.**

|  | **BC9F** | **BC13F** | **BC12M** | **BC13M** | **UC12F** | **UC14F** | **UC10M** | **UC13M** |
| --- | --- | --- | --- | --- | --- | --- | --- | --- |
| **BC9F** |  |  |  |  |  |  |  |  |
| **BC13F** | 0.60** |  |  |  |  |  |  |  |
| **BC12M** | 0.44* | 0.85** |  |  |  |  |  |  |
| **BC13M** | 0.44* | 0.85** | 0.66** |  |  |  |  |  |
| **UC12F** | 1.00*** | 1.00*** | 1.00*** | 1.00*** |  |  |  |  |
| **UC14F** | 0.99*** | 1.00*** | 1.00*** | 1.00*** | 1.00*** |  |  |  |
| **UC10M** | 0.99*** | 1.00*** | 1.00*** | 1.00*** | 1.00*** | 1.00*** |  |  |
| **UC13M** | 1.00*** | 1.00*** | 1.00*** | 1.00*** | 1.00*** | 1.00*** | 1.00*** |  |

*** p < 0.002 ; ** p < 0.01; * p < 0.05
